# Regenerative Potential of Biodentine in Complex Endodontic Conditions: A Systematic Review of Clinical and Radiological Evidence

**DOI:** 10.3390/medicina62071321

**Published:** 2026-07-08

**Authors:** Alexandra Mihaela Stoica, Liana Bereșescu, Monica Dana Monea, Timea Dakó, Alexandru Vlasa, Csilla Benedek, Oana Elena Stoica, Mahmoud Saafin, Cristina Stanca Molnar Varlam

**Affiliations:** Faculty of Dental Medicine, George Emil Palade University of Medicine, Pharmacy, Science, and Technology of Târgu Mureș, 38 Gheorghe Marinescu Str., 540139 Târgu Mureș, Romania; alexandra.stoica@umfst.ro (A.M.S.); monica.monea@umfst.ro (M.D.M.); timea.dako@umfst.ro (T.D.); alexandru.vlasa@umfst.ro (A.V.); csilla.benedek@umfst.ro (C.B.); oana.stoica@umfst.ro (O.E.S.); nabil.elsaafinmahmoud@umfst.ro (M.S.); cristina.molnar@umfst.ro (C.S.M.V.)

**Keywords:** Biodentine, biomaterials, endodontics, bone regeneration, bone healing

## Abstract

*Background/Objectives:* Complex endodontic lesions characterized by significant periapical bone loss, diverse anatomical variations in the root canal system and apical resorption represent a major therapeutic challenge. Biodentine, a calcium silicate-based bioactive dental restorative material, has gained considerable attention because of its potential to promote and sustain the regeneration of bone tissue. This review aims to evaluate current evidence on Biodentine’s regenerative abilities in treatments of diverse endodontic pathology and highlight the clinical and radiographic outcomes. *Material and Methods:* A systematic review was conducted in accordance with PRISMA guidelines by searching for articles in three electronic databases: Medline (PubMed), Scopus, and Cochrane Library. Studies describing the application of Biodentine in cases of complex endodontic pathology with destruction of apical bone and apical resorption of roots were considered for inclusion in the study. Quality assessment was carried out using the Cochrane risk of bias assessment tool RoB 2.0. *Results:* The included clinical and radiographic studies demonstrated positive treatment outcomes after using Biodentine in difficult endodontic lesions, including a reduction in lesion size, improvement in symptoms and progression of periapical bone regeneration after 12 months of follow-up. No significant adverse outcomes were reported in the studies included. *Conclusions:* Biodentine proved to be an efficient biocompatible material in terms of managing complex endodontic lesions. Due to its bioactive properties and high efficiency as an apical plug, Biodentine is capable of inducing bone regeneration within the affected periapical area.

## 1. Introduction

Endodontic infections represent one of the most common causes of inflammatory processes in periapical bone and ligament tissue. Pathological changes develop because of the colonization of the root canal system by bacteria, which provokes the inflammation and destruction of bone structures. Under the influence of bacterial toxins and by-products, periapical lesions form, which radiographically correspond to areas of osteolysis due to osteoclast activity in the proximity of the root of the affected tooth [1,2].

Depending on the complexity, lesion size, and type of pathological change, there may be different symptoms of periapical disease. Small periapical lesions may be resolved after endodontic treatment; however, in case of complicated endodontic lesions, such as large periapical bone destruction, root internal and external resorptions, perforations, chronic intraarticular infection, or failure of previous endodontic treatment, extra difficulties are presented in the healing process [3,4].

The goal of any endodontic treatment is the elimination of infection, prevention of reinfection, and creation of an appropriate biological environment for regeneration of periapical tissues. The successful result of treatment is characterized by inflammation resolution and periapical bone structure regeneration. In the cases of complicated endodontic lesions, spontaneous bone regeneration may not occur or may take place incompletely, therefore requiring the development of additional approaches to facilitate periapical tissue regeneration [5].

During the last few decades, numerous bioactive materials have been created to help achieve positive outcomes in endodontic treatment. As the name suggests, bioactive materials may interact with biological tissues and trigger a series of biological responses, which are favorable for further tissue restoration and healing. Bioactive materials are able to liberate ions with biological activity, stimulate mineralization processes, and create a proper environment for tissue healing [6].

Among the bioactive materials available, calcium silicate-based cements are rather popular because of their excellent biological properties and tissue regeneration capability. Biodentine represents a tricalcium silicate-based bioactive material, which was designed as a dentine substitute and applied clinically in various treatments in dentistry and endodontics. The material possesses excellent physical properties, such as good compressive strength, high degree of sealing, and dimensional stability, which make it suitable for procedures involving replacement and sealing of root canals and dentine [7].

Moreover, Biodentine demonstrates favorable biological properties, which contribute to tissue regeneration. Experimental and clinical studies confirm that Biodentine has a great degree of biocompatibility and stimulates mineralization and formation of hard tissue. The material is capable of promoting the release of dentine matrix growth factors and odontoblast-like cell differentiation, which stimulates hard tissue regeneration and mineralization. Moreover, Biodentine has the potential to stimulate osteogenesis and bone tissue formation in periapical regions [8,9].

It is worth noting that another biological characteristic of Biodentine is the possibility of creating an alkaline environment, which has antimicrobial properties. The creation of such an alkaline environment promotes hydroxyapatite formation at the surface of dentine and contributes to creation of a good seal between material and dentine surface [10].

There are plenty of clinical studies on positive clinical outcomes of treating complex endodontic pathologies, such as large periapical lesions, root perforations, and root resorptions. Numerous cases show that Biodentine application resulted in periapical bone regeneration and successful periapical healing in radiographs [11,12]. Despite the increasing amount of evidence on the clinical effectiveness of Biodentine, the information found in the literature is rather heterogeneous, with a great variety of research designs, study samples, and methods of evaluation [13,14]. For the purpose of the present review, the term “complex endodontic lesions” refers to clinical situations characterized by extensive periapical bone destruction, apical periodontitis associated with large radiolucent lesions, external or internal root resorption, root perforations, persistent apical pathology after previous endodontic treatment, and regenerative endodontic cases requiring bioactive materials to promote healing and tissue regeneration. These conditions are generally associated with a less predictable prognosis and require advanced treatment approaches beyond conventional root canal therapy.

Therefore, a systematic review of the literature should be carried out to understand the current state of science and determine whether there is sufficient evidence of Biodentine’s potential in complicated endodontic lesions. The purpose of the systematic review is to provide a critical analysis and synthesis of all the available scientific evidence on the use of Biodentine in endodontic lesions, with particular consideration paid to clinical and radiological findings. The aim of this systematic review was to evaluate the clinical, radiographic, and regenerative outcomes associated with Biodentine in the management of complex endodontic conditions. Particular emphasis was placed on evidence related to periapical healing, hard tissue formation, and tissue regeneration, while acknowledging that the included studies addressed different clinical indications and levels of evidence regarding periapical bone regeneration.

## 2. Materials and Methods

The present systematic review was conducted to answer the main guiding research question: “What is the potential of Biodentine to promote bone regeneration in the management of complex endodontic lesions?” The purpose of the review was to assess scientific data on the regenerative potential of Biodentine when dealing with endodontic problems connected with periapical bone destruction.

The research strategy involved systematic searching and analysis of the literature devoted to Biodentine application in the treatment of complex cases of endodontic lesions, such as large periapical lesions, root resorption, root canal perforation, and chronic periapical infections. Particular emphasis was placed on the results concerning periapical recovery and bone regeneration after Biodentine therapy. The review study was conducted in accordance with the PRISMA 2020 Statement. The completed PRISMA 2020 Checklist is available in the Supplementary Materials (Supplementary File S1).

### 2.1. Data Sources and Search Strategy

Comprehensive research of the medical literature was performed using three major electronic databases, namely, Medline through PubMed, Scopus, and Cochrane Library. The reason for this was to get articles about the effect of Biodentine on bone regeneration and the endodontic protocol.

In performing a comprehensive search, Medical Subject Headings (MeSH terms) and keywords were used to cover all the possible articles that could be considered relevant. These keywords are:

- Biodentine AND bone regeneration;
- Biodentine AND periapical lesions;
- Biodentine AND endodontic treatment;
- Biodentine AND periapical healing.

Boolean operators were used to refine the search strategy and improve the accuracy of all the results that were obtained. The search included all studies published until March 2026, without any kind of restrictions regarding the year of publication. Only articles written in English and involving human subjects were used and considered eligible for screening. All records were exported and screened for duplicates before proceeding with the study selection process. The study selection process is summarized in Table 1, presenting the number of records identified, screened, excluded, and included in the systematic review according to PRISMA guidelines.

### 2.2. Study Selection and Methodology for Data Collection

A preliminary database search using different keywords included 161 articles. In order to narrow down the literature database and filter out studies related to our topic of interest, a three-stage study selection method was followed based on the established criteria (Table 2).

**Figure 1:**
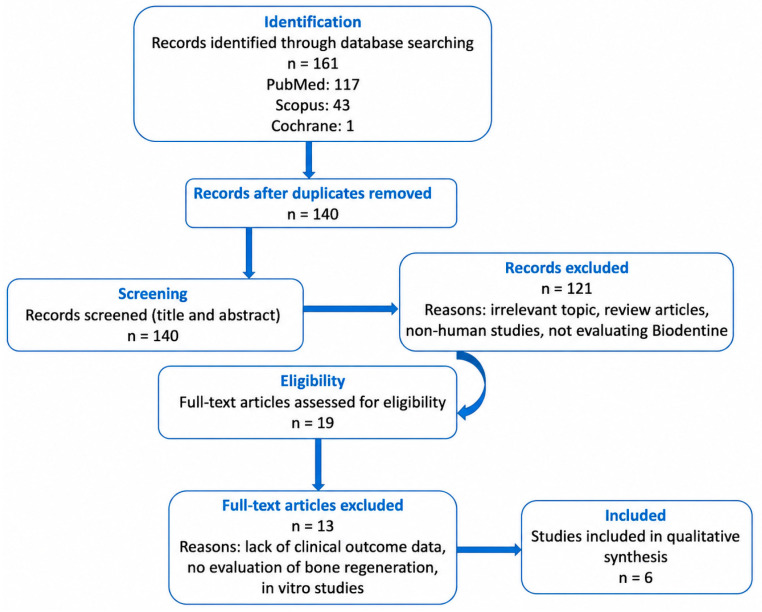
Flowchart conducted during the methodology.

Following the application of the predefined inclusion and exclusion criteria, the titles and abstracts of all retrieved records were independently screened by two reviewers (A.M.S. and L.B.) to identify studies relevant to the objective of the present systematic review. Studies considered potentially eligible during the initial screening stage were subsequently subjected to a full-text assessment by the same reviewers according to the predefined eligibility criteria. Any disagreement regarding study eligibility was resolved through discussion and consensus. When consensus could not be reached, a third reviewer (M.D.M.) was consulted to make the final decision. The study selection process was conducted in accordance with PRISMA recommendations to ensure methodological transparency and reproducibility. Following the full-text evaluation, six studies were identified as meeting all eligibility criteria and were included in the qualitative synthesis of this systematic review. Therefore, 6 studies were identified as directly addressing the role of Biodentine in the management of complex endodontic lesions and bone regeneration and were included in the qualitative synthesis of this systematic review (Figure 1).

### 2.3. Data Extraction and Data Synthesis

Extraction of the data was performed independently by two reviewers utilizing a predefined data extraction form. Information regarding study design, sample characteristics, clinical indication, Biodentine application protocol, comparator intervention, follow-up duration, and reported clinical and radiographic outcomes was extracted from all included studies. The extracted characteristics of the included studies are summarized in Table 3. When detailed clinical parameters such as lesion size, CBCT characteristics, thickness of Biodentine, or irrigation protocol were not available in the original publication, these items were recorded as not reported.

### 2.4. Risk of Bias Assessment

Methodological quality and risk of bias for the included clinical trials were evaluated independently by two reviewers using the Cochrane risk of bias tool (RoB 2.0). This assessment tool enables the structured evaluation of the methodological quality of clinical trials and aids in judging whether the outcomes observed in these clinical trials with regard to periapical healing and bone regeneration after treatment using Biodentine are reliable.

Some of the domains considered while evaluating for risk of bias included:

○ Bias arising from all randomization processes;
○ Bias due to deviations from intended interventions;
○ Bias due to incomplete or missing outcome data;
○ Bias from selective outcome measurement and clinical outcomes;
○ Bias in selective outcome reporting of all results.

Each of the domains was assessed using predetermined standards, which categorized them as low risk of bias, some concerns, or high risk of bias.

In case of any disagreement among the reviewers, the issue was resolved by discussion until a consensus was reached, and if needed, the judgment was made by an independent third party. The outcomes of the risk of bias analysis were considered while interpreting the data.

## 3. Results

A total of 161 records were identified through database searching. After duplicate removal and title and abstract screening, 19 full-text articles were assessed for eligibility. Of these, 13 studies were excluded based on predefined criteria, and ultimately, six clinical studies were included in the qualitative synthesis of this systematic review. Of the six included studies, three directly investigated conditions involving periapical lesions, apical periodontitis, or regenerative endodontic procedures and therefore provided direct evidence regarding periapical healing. The remaining three studies evaluated pulpotomy or indirect pulp capping procedures and were included because they provided indirect evidence regarding the biological and regenerative properties of Biodentine. The outcomes reported in the included studies primarily focused on clinical improvement and radiological evidence of periapical bone healing during follow-up periods ranging from several months to multiple years. The following table summarizes the most important findings of the present research (Table 4).

For the purpose of this review, treatment success was considered according to the criteria reported in the included studies as follows: (1) absence of clinical symptoms such as pain, swelling, or tenderness to percussion; (2) maintenance or recovery of tooth function; (3) radiographic evidence of periapical healing, including reduction in lesion size, progressive bone fill, resolution of radiolucency, or formation of mineralized tissue. These three parameters were used for the comparison of all outcomes presented in studies.

Of the included articles, five (83.3%) were randomized controlled trials, while only one (16.7%) article included clinical comparative research, signifying an overall moderate-to-high level of evidence among the included studies. Biodentine was used for various indications, such as pulpotomy, intracanal dressing, apical barrier creation, and regeneration procedures in endodontics. Follow-up time periods also differed between articles, ranging from short follow-up to 18 months. This factor should be taken into account regarding long-term evaluation.

In spite of the differences in methodology, clinical indication, and application protocol, all results were proven to be positive among the included papers. The majority of research demonstrated a lack of clinical symptoms, such as pain and swelling, and the return of tooth functionality. In terms of radiographic evaluation, there is a tendency towards lesion regression and bone repair in apical areas.

Nevertheless, heterogeneity in population characteristics, methodology, and evaluation criteria of results needs to be accounted for. An important aspect to consider is that not all included studies directly investigated periapical bone regeneration associated with complex endodontic lesions. Three studies specifically evaluated conditions involving apical periodontitis, periapical lesions, or regenerative endodontic procedures and therefore provided direct evidence regarding periapical healing. The remaining studies investigated pulpotomy or indirect pulp capping procedures and were included because they evaluated the biological and regenerative properties of Biodentine in clinical settings. Consequently, these studies provide indirect evidence supporting the regenerative potential of Biodentine and should be interpreted with caution when extrapolating conclusions regarding periapical bone regeneration.

## 4. Discussion

This systematic review aimed to assess the regenerative capabilities of Biodentine in treating complex endodontic lesions with particular emphasis on clinical and radiographic results. According to the findings of all included articles, the usage of Biodentine was found to result in positive therapeutic outcomes characterized by an improvement in patients’ conditions and periapical tissue healing [14,15]. The interpretation of the findings should take into account that the included studies addressed different clinical indications. While studies evaluating regenerative endodontic procedures and apical periodontitis directly assessed periapical healing outcomes, studies investigating pulpotomy and indirect pulp capping primarily evaluated pulp healing and dentin bridge formation. Therefore, the available evidence is not entirely homogeneous, and conclusions regarding periapical bone regeneration are supported mainly by a subset of the included studies.

Positive clinical outcomes (i.e., pain reduction, resolution of inflammation, and maintenance of tooth function), together with favorable radiographic findings reported in several studies, suggest that Biodentine may be associated with beneficial therapeutic outcomes in selected endodontic conditions [16,17]. However, the interpretation of these findings should consider the heterogeneity of the included studies and the limited number of investigations directly evaluating periapical bone healing. These findings are in line with existing evidence on the bioactivity of calcium silicate-based materials and their effectiveness in treating different conditions [14,18]. A critical interpretation of the available evidence is necessary because the included studies demonstrated substantial methodological and clinical heterogeneity. The investigated populations ranged from pediatric patients undergoing pulpotomy procedures to adult patients presenting with apical periodontitis, pulp necrosis, or regenerative endodontic treatment needs. Biodentine was used for different clinical purposes, including pulpotomy, indirect pulp capping, intracanal medication, cervical barriers in regenerative procedures, and apical plug formation. Moreover, follow-up periods varied considerably, ranging from one week to eighteen months, while outcome assessment methods included clinical evaluation, radiographic healing, histological analysis, magnetic resonance imaging, and measurement of inflammatory biomarkers. Such heterogeneity limits direct comparisons among studies and reduces the ability to draw definitive conclusions regarding the specific role of Biodentine in periapical bone regeneration [19,20].

The favorable clinical and radiographic outcomes reported in the included studies may be partially explained by the biological properties of Biodentine. First, it releases Ca++ ions and provides an alkaline pH, which favors tissue mineralization [20,21]. Second, the material stimulates differentiation of odontoblast-like cells and enhances the process of both dentinogenesis and osteogenesis. The third factor favoring tissue regeneration and healing is its excellent sealing properties, which prevent bacterial reinfiltration [22,23,24]. Several of the included studies compared Biodentine with MTA or other calcium silicate-based materials and generally reported favorable clinical outcomes for both materials. However, these findings should be interpreted cautiously because the studies differed substantially in terms of clinical indications, treatment protocols, outcome measures, and follow-up periods. Therefore, direct comparisons between Biodentine and alternative biomaterials remain limited, and the currently available evidence does not allow definitive conclusions regarding the superiority of one material over another to be drawn. Future comparative clinical trials using standardized protocols are needed to better establish the relative effectiveness of these materials [25,26,27].

Another valuable characteristic of Biodentine, which is highlighted by all reviewed studies, is its versatility. It can be applied in pulpotomy, intracanal dressing, apical barrier formation, and regenerative endodontics, and each of these applications is followed by positive treatment outcomes. These facts show that Biodentine may be considered an effective alternative to conventional MTA in a wide range of clinical cases [18,28].

The findings of the present review should also be interpreted within the broader context of calcium silicate-based biomaterials currently used in endodontics. Recent prospective clinical studies by Spinelli et al. reported favorable clinical and radiographic outcomes following root canal treatment performed with premixed bioceramic sealers and different obturation techniques [29,30,31]. However, these studies investigated root canal sealers used in conventional endodontic treatment protocols and did not specifically evaluate Biodentine or its application in the management of complex endodontic lesions. Therefore, their findings cannot be directly compared with the studies included in the present review, which assessed Biodentine in different clinical applications such as regenerative endodontic procedures, apical barrier formation, pulpotomy, or indirect pulp capping [32]. These studies are discussed only to provide a broader context regarding the clinical performance of calcium silicate-based biomaterials and should not be considered direct evidence supporting the regenerative potential of Biodentine. Further comparative studies are required to clarify the relative clinical performance of different calcium silicate-based materials in specific endodontic indications.

When interpreting the presented outcomes, we also took into consideration the potential influence of methodological bias. The included studies presented differences in study design, sample size, follow-up duration, and outcome assessment methods. Moreover, studies reporting positive outcomes are more likely to be published, thus leading to a potential publication bias. Therefore, although the available evidence consistently suggests favorable clinical and radiographic outcomes associated with Biodentine, the overall strength of the evidence should be studied with caution.

However, it is necessary to pay attention to some limitations of this study. For instance, its results are based on a relatively limited number of included studies; hence, further research is required in order to draw stronger conclusions about Biodentine. Second, included articles exhibit significant heterogeneity; thus, no quantitative analysis could have been performed, and a direct comparison between the studies was difficult. Furthermore, the length of follow-up differed significantly, and most studies only provided data for the mid-term period after treatment, which raises questions regarding its long-term effects. Finally, even though the majority of the papers included belonged to RCTs, their heterogeneity and potential sources of bias should not be neglected.

From a clinical perspective, the findings of this systematic review demonstrate the ability of Biodentine to regenerate tissues affected by various endodontic complications due to its beneficial biological properties, convenient handling, and excellent sealing properties. Thus, it may be regarded as a promising bioactive material for treating complex lesions in teeth.

The next step that needs to be taken is designing well-conducted randomized controlled trials based on standard treatment protocols and involving a large number of subjects. In addition, the follow-up period should cover a long enough time period in order to assess the real long-term effects of Biodentine use. Also, the employment of modern radiographic techniques, such as CBCT scanning, would provide a more reliable evaluation of bone regeneration. Although Biodentine has shown promising clinical and radiographic outcomes in the management of complex endodontic lesions, further research is needed to clarify its long-term effectiveness and to better establish its role in tissue healing and regeneration.

## 5. Conclusions

Within the limitations of the present systematic review, Biodentine appears to be associated with favorable clinical and radiographic outcomes in the management of selected endodontic conditions, particularly in cases involving periapical pathology, regenerative endodontic procedures, and apical barrier formation. Its bioactivity, sealing ability, and biocompatibility may contribute to tissue healing and treatment success. However, the current evidence base remains limited due to the heterogeneity of the included studies, the small number of directly relevant investigations, variable sample sizes, and relatively short follow-up periods. Furthermore, several included studies provided only indirect evidence regarding periapical bone healing.

Therefore, while Biodentine represents a promising biomaterial in endodontic practice, definitive conclusions regarding its ability to induce periapical bone regeneration cannot yet be established. Additional high-quality randomized clinical trials with standardized methodologies and extended follow-up periods are required to strengthen the available evidence.

## Figures and Tables

**Table 1:** PRISMA flow diagram data.

Stage	Description	Number (n)
Identification	Records identified through database searching	161
	PubMed	117
	Scopus	43
	Cochrane	1
	Records after duplicates removed	140
Screening	Records screened (title and abstract)	140
	Records excluded (Reasons: irrelevant topic, review articles, non-human studies, no evaluation of Biodentine)	121
Eligibility	Full-text articles assessed for eligibility	19
	Full-text articles excluded (Reasons: lack of clinical outcome data, no evaluation of bone regeneration, in vitro studies)	13
Included	Studies included in qualitative synthesis	6

**Table 2:** Inclusion criteria for study selection.

Criterion Category	Inclusion Criteria	Exclusion Criteria
Publication Date	No restriction regarding the year of publication	—
Study Population	Studies conducted on human subjects	Studies conducted on animals or in vitro studies
Journal Category	Articles published in journals related to dentistry and oral health	Articles published in non-relevant medical or non-scientific journals
Study Design	Original clinical studies (clinical trials, case series, case reports)	Review articles, editorials, letters to the editor
Intervention	Studies investigating the clinical use of Biodentine in endodontic treatments associated with tissue healing, hard tissue formation, regenerative outcomes, or periapical healing.	Studies not evaluating Biodentine or not assessing clinical outcomes
Outcomes	Studies reporting clinical, radiographic, histological, or biological outcomes related to tissue healing, mineralized tissue formation, regenerative responses, or periapical healing.	Studies lacking clinical outcome data
Language	Articles published in English	Articles published in languages other than English

**Table 3:** Characteristics of the studies included in the qualitative synthesis and extracted variables.

Author (Year)	Study Design	Sample	Clinical Condition	Biodentine Application	Comparator	Follow-Up	Extracted Outcomes
Guang et al. (2022) [14]	Randomized comparative clinical study	60 primary molars (30/group)	Primary molars requiring pulpotomy	Pulpotomy material	Formocresol	12 months	Clinical success, radiographic success, inflammation, necrosis
Chhabra et al. (2025) [15]	Randomized controlled trial	68 children; 100 molars analyzed	Deep carious primary molars with reversible pulpitis	Indirect pulp capping material	MTA	18 months	Clinical success, radiographic success, pulp vitality
Ünal et al. (2025) [16]	Randomized controlled clinical trial	60 patients	Asymptomatic apical periodontitis	Tricalcium silicate intracanal dressing	Calcium hydroxide	7 days	RANKL/OPG, TNF-α, PGE-2, TGF-β
Al-Rawhani et al. (2024) [17]	Randomized controlled clinical trial	36 patients (31 completed)	Pulp necrosis and apical periodontitis	Coronal barrier in REP	MTA	18 months	Periapical healing, tooth sensibility
El-Kateb et al. (2020) [18]	Randomized controlled clinical trial	18 mature necrotic teeth	Necrotic teeth with periapical lesions	Cervical plug in REP	X3 vs. X5 apical preparation	12 months	MRI tissue regeneration, lesion healing, sensibility
Nassri et al. (2026) [19]	Randomized double-blind histological trial	60 premolars from 40 patients	Symptomatic irreversible pulpitis	Full pulpotomy material	MTA and TotalFill BC RRM	1 week and 6 months	Histological healing, inflammatory response

**Table 4:** Summary of clinical and radiological outcomes of Biodentine in the management of complex endodontic lesions.

No.	Author (Year)	Clinical Outcome	Radiological Outcome
1	Guang et al., 2022 [14]	Absence of pain, swelling, or fistula	Radiographic success rate of 93.9% at 12 months
2	Ünal et al., 2025 [15]	Significant reduction in inflammatory mediators	Reduction in bone resorption markers
3	Nassri et al., 2026 [16]	Reduction in inflammation and pulp healing	Formation of dentinal-like bridge
4	Chhabra et al., 2025 [17]	Clinical success rate of 96%	Radiographic success rate comparable to MTA
5	Al-Rawhani et al., 2024 [18]	Resolution of symptoms and regained sensibility	Healing of periapical lesions in majority of cases
6	El-Kateb et al., 2020 [19]	All treated teeth became symptom-free	Complete radiographic healing of periapical lesions

## Supplementary Materials

The following are available online at https://www.mdpi.com/article/10.3390/medicina62071321/s1, Table S1: PRISMA checklist.

## References

[1] Thiyagarajan G., Manoharan M., Veerabadhran M.M., Murugesan G., Vinodh S., Kamatchi M. (2023). Biodentine as BioRoot inlay: A case report. Int. J. Clin. Pediatr. Dent..

[2] Al-Ahmad A., Haendel M., Altenburger M.J., Karygianni L., Hellwig E., Wrbas K.T., Vach K., Tennert C. (2022). Biodentine inhibits the initial microbial adhesion of oral microbiota in vivo. Antibiotics.

[3] Subhi H., Subhi N., Alhaidary S., Azeez M.S., Tabnjh A.K. (2025). Antibacterial activity of Biodentine against Enterococcus faecalis: A systematic review. Front. Dent. Med..

[4] Wang X., Cai Y., Zhang M., Xu J., Zhang C., Li J. (2022). Effect of Biodentine on odonto/osteogenic differentiation of human dental pulp stem cells. Bioengineering.

[5] Riandani A.P., Cahyanto A., Diab R.A.L., Widyasari R., Sidiqa A.N., Dharsono H.D.A., Zakaria M.N. (2025). Clinical and radiographic outcomes of pulpotomy materials in permanent teeth: A systematic review of calcium hydroxide, MTA, Biodentine, and iRoot BP Plus. BMC Oral Health.

[6] Lu K.Y., Gibbs J.L., Wu C.Y., Blatz M.B., Ma X., Fu M.W., Ma K.S. (2025). Efficacy of Biodentine versus mineral trioxide aggregate in pulpotomy for primary teeth: A systematic review and meta-analysis of randomized controlled trials. J. Evid. Based Dent. Pract..

[7] Brizuela M., Daley J.O. (2025). Dental Materials: Biodentine, a Calcium Silicate Bioactive.

[8] Kunam D., Bode Y., Narra P.M., Krishna C.H.N.V.M., Reddy S.N., Venkateshwarlu M. (2024). Comparative evaluation of apical microleakage of mineral trioxide aggregate, Biodentine, and Bio-C Repair as root-end filling materials using dye extraction method: An in vitro study. J. Conserv. Dent. Endod..

[9] Paranna S., Gogawale R., Patil A., Huddar S., Patro K., Kanetkar J. (2025). Clinical and radiographic evaluation of Biodentine for apexogenesis in young permanent mandibular molars: A systematic review and meta-analysis. Cureus.

[10] Zamparini F., Prati C., Taddei P., Spinelli A., Di Foggia M., Gandolfi M.G. (2022). Chemical-physical properties and bioactivity of new premixed calcium silicate-bioceramic root canal sealers. Int. J. Mol. Sci..

[11] Halder N., Vemuri S., Guptha Anila B.S., Bolla N., Garlapati R., Basam R.C. (2023). To compare the efficacy of various organic solvents on retrievability of Biodentine and their effect on microhardness of Biodentine and radicular dentin: An in vitro study. J. Conserv. Dent..

[12] Eraković M., Bekić M., Đokić J., Tomić S., Vučević D., Pavlović L., Duka M., Marković M., Bokonjić D., Čolić M. (2025). Biodentine stimulates calcium-dependent osteogenic differentiation of mesenchymal stromal cells from periapical lesions. Int. J. Mol. Sci..

[13] Tran X.V., Ngo L.T.Q., Boukpessi T. (2021). Biodentine full pulpotomy in mature permanent teeth with irreversible pulpitis and apical periodontitis. Healthcare.

[14] Guang J., Li J., Hao L. (2022). Clinical observation and histopathological evaluation of pulp after pulpotomy of primary teeth with formocresol and Biodentine. Cell. Mol. Biol..

[15] Ünal O., Sümbüllü M., Laloğlu E. (2025). Effect of tricalcium silicate-based intracanal dressing on bone resorption and inflammatory mediators in periapical lesions: A randomized controlled clinical trial. Odontology.

[16] Nassri S., Mardini A., Aljabban O., Tolibah Y.A. (2026). Histological evaluation of vital full pulpotomy techniques in permanent mature teeth with symptomatic irreversible pulpitis using three calcium silicate cements: A randomized controlled clinical trial. BMC Oral Health.

[17] Chhabra N., Chhabra A., Mehta R., Bains S., Mauli G. (2025). Clinical and radiographic evaluation of Biodentine and mineral trioxide aggregate as indirect pulp capping agents in primary molars: A randomized controlled trial with 18-month follow-up. Ann. Afr. Med..

[18] Al-Rawhani A.H., Mohamed Ibrahim S., Mohamed Abu Naeem F. (2024). Regenerative treatment of mature teeth with pulp necrosis and apical periodontitis using Biodentine compared with MTA: Randomized controlled clinical trial. Eur. Endod. J..

[19] El-Kateb N.M., El-Backly R.N., Amin W.M., Abdalla A.M. (2020). Quantitative assessment of intracanal regenerated tissues after regenerative endodontic procedures in mature teeth using magnetic resonance imaging: A randomized controlled clinical trial. J. Endod..

[20] Rajasekharan S., Martens L.C., Cauwels R.G.E.C., Verbeeck R.M.H. (2014). Biodentine™ material characteristics and clinical applications: A review of the literature. Eur. Arch. Paediatr. Dent..

[21] Laurent P., Camps J., About I. (2012). Biodentine™ induces TGF-β1 release from human pulp cells and early dental pulp mineralization. Int. Endod. J..

[22] Nowicka A., Lipski M., Parafiniuk M., Sporniak-Tutak K., Lichota D., Kosierkiewicz A., Kaczmarek W., Buczkowska-Radlińska J. (2013). Response of human dental pulp capped with Biodentine and mineral trioxide aggregate. J. Endod..

[23] Camilleri J. (2013). Investigation of Biodentine as dentine replacement material. J. Dent..

[24] Koubi G., Colon P., Franquin J.C., Hartmann A., Richard G., Faure M.O., Lambert G. (2013). Clinical evaluation of the performance and safety of a new dentine substitute, Biodentine, in the restoration of posterior teeth—A prospective study. Clin. Oral Investig..

[25] Zanini M., Balice G., Kérourédan O., Lézé C., Gueyffier F., Maucort-Boulch D., Nony P., Grosgogeat B., Pradalle N. (2025). Outcomes of Direct Pulp Capping After Carious Excavation of Deep Caries on Permanent Mature Teeth: An Ancillary Study Derived From A Randomised Clinical Trial. Eur. Endod. J..

[26] Menteş İ., Sümbüllü M. (2025). Effect of tricalcium silicate-based intracanal dressing on antibacterial-antifungal activity and postoperative pain intensity after non-surgical endodontic retreatment: Randomized controlled clinical trial. Head. Face Med..

[27] Srinivasan D., Narayan S., Mungara J., Shreya S. (2025). Evaluation of silver diamine fluoride and biodentine as agents for indirect pulp therapy in primary teeth—A randomized controlled trial. J. Indian Soc. Pedod. Prev. Dent..

[28] Wadhwa H., Duhan J., Sangwan P., Tewari S., Kumar V., Mittal S., Arora M. (2025). Effect of Age on the Success of Direct Pulp Capping using 2 Bioceramic Materials in Cariously Exposed Teeth with Reversible Pulpitis: A Prospective Clinical Study. J. Endod..

[29] Deshmukh S.N., Shenoy V.U., Margasahayam S.V., Chaudhri G.U. (2024). Comparative Evaluation of Efficacy of Resin-modified Glass Ionomer Cement and Light-curable Tricalcium Silicate Cement as Indirect Pulp Capping Materials: A Randomized Clinical Trial. J. Contemp. Dent. Pract..

[30] Oburo F.O., Adegbulugbe I.C., Awotile A.O., Enone L.L., Oyapero A. (2024). Evaluation of Biodentine^®^ and Calcium Hydroxide in the Formation of Dentin Bridge in Deep Carious Lesions. West Afr. J. Med..

[31] Spinelli A., Zamparini F., Lenzi J., Gandolfi M.G., Prati C. (2023). Clinical Evaluation of a Novel Premixed Tricalcium Silicate Containing Bioceramic Sealer Used with Warm Carrier-Based Technique: A 12-Month Prospective Pilot Study. Appl. Sci..

[32] Spinelli A., Zamparini F., Lenzi J., Gandolfi M.G., Prati C. (2024). Three-year Clinical Outcome of Root Canal Treatment Using a Single-cone Technique and Ceraseal Premixed Bioceramic Sealer: A Prospective Cohort Study. Eur. Endod. J..

